# Detection of SARS-CoV-2 N501Y mutation among SARS-CoV-2 variants of concern circulating in Northern Cyprus

**DOI:** 10.2217/fvl-2021-0273

**Published:** 2022-06-08

**Authors:** Gulten Tuncel, Mahmut Cerkez Ergoren, Buket Baddal, Pinar Tulay, Cenk Serhan Ozverel, Emrah Guler, Huseyin Kaya Suer, Murat Sayan, Tamer Sanlidag

**Affiliations:** ^1^DESAM Research Institute, Near East University, Nicosia, 99138, Cyprus; ^2^Department of Medical Genetics, Faculty of Medicine, Near East University, Nicosia, 99138, Cyprus; ^3^Department of Clinical Microbiology & Medical Microbiology, Faculty of Medicine, Near East University, Nicosia, 99138, Cyprus; ^4^Department of Basic Medical Sciences, Faculty of Dentistry, Near East University, Nicosia, 99138, Cyprus; ^5^Faculty of Health Sciences, Near East University, Nicosia, 99138, Cyprus; ^6^Department of Infectious Diseases & Clinical Microbiology, Faculty of Medicine, Near East University, Nicosia, 99138, Cyprus; ^7^PCR Unit, Educational & Research Hospital of Kocaeli University, Kocaeli University, Kocaeli, 41001, Turkey

**Keywords:** B.1.1.7, B.1.351, COVID-19, N5017Y, P.1, SARS-CoV-2, variants of concern

## Abstract

**Aim:** SARS-CoV-2 variants of concern (VOCs) carry signature mutations particularly in the spike protein. Most VOCs lineages that carry N501Y substitution have been reported to evade viral diagnostic tests and have impact on vaccine effectiveness. Therefore, monitoring the circulating variants represents a major requirement for a public health response worldwide. We aimed to investigate the prevalence of N501Y bearing SARS-CoV-2 samples in Northern Cyprus. **Materials & methods:** Reverse transcription quantitative PCR technique was used to identify N501Y mutation from 658 samples. **Results:** Our results indicate that the proportion of N501Y-bearing lineages increased significantly from January through May 2021 (45.2–75.5%) in the region. **Conclusion:** These results indicate that VOCs are dominant lineages in the country and highlight an alarming situation which require strict governmental measures to minimize COVID-19 morbidity and mortality.

The importance of genetic analyses of the SARS-CoV-2 has been proven in the development of sensitive diagnostics and effective treatment strategies. Since the emergence of the COVID-19 pandemic, these analyses have been used to identify sequence variations and provide critical data in in the control of strains with higher transmissibility and vaccine resistance.

First complete genetic sequence of SARS-CoV-2 was obtained via deep meta-transcriptomic sequencing of the complementary DNA obtained from the RNA sample extracted from bronchoalveolar lavage fluid of a patient who had typical symptoms of COVID-19 including fever, chest tightness, cough and pain and who was hospitalized in Central Hospital of Wuhan on 26 December 2019 [1]. Since then, several variants have been reported that could possibly lead to changes in transmissibility, clinical presentation and severity of the cases worldwide.

In December 2020, authorities of the UK reported a variant which was initially detected in South East England from a specimen dated 20 September 2020 and took over other virus lineages across the UK within a few weeks [2,3]. This new variant was referred to as SARS-CoV-2 variant of concern (VOC) 202012/01, B.1.1.7, later as the Alpha variant and contains 23 nucleotide substitutions, nine of which are within the spike (S) protein. Among these, substitution at position 501, where an arginine residue is replaced with a lysine residue (N501Y), attracted attention as it was shown to increase binding of the virus to host ACE2 receptor and increase cell infectivity in transgenic mice models expressing human ACE2 protein [4,5]. Therefore, N501Y mutation is considered to be responsible for the higher transmissibility reported for the B.1.1.7. lineage. The variant also possesses a deletion at amino acid positions 69 and 70 of the S protein (S dropout), which was reported to be associated with reduced test sensitivity and molecular or antigen-based diagnostic test failures [6]. Hence, such variants with high transmissibility rates, which could escape from initial diagnostic tests, have the potential to spread rapidly from its emerging location to several other countries within a few months through travel.

Shortly after the detection of the B.1.1.7. variant in the UK, N501Y mutation was also detected in other emerging SARS-CoV-2 lineages that were reported in South Africa (B.1.351, Beta) and Brazil (P.1, Gamma) [7,8]. Both of these variants, termed VOCs, have increased transmissibility, resulting in the rapid displacement of other pre-existing lineages.

There are studies emphasizing cross-species transmission of N501Y-bearing SARS-CoV-2 variants. Study by Li *et al.* indicated that the S proteins of Alpha, Beta and Gamma variants endowed increased infectivity especially in cells expressing mouse ACE2, which was likely associated with N501Y, E484K and K417N mutations in the receptor binding domain (RBD) [9]. In agreement, another study showed that N501Y mutation alone could render effective binding of the spike protein to mouse ACE2 receptors, leading to effective infection; while the wild-type strain could not bind to mouse ACE2 to mediate membrane fusion and virus infection [10,11]

While most countries are building or upscaling high-throughput sequencing capacities, whole genome sequencing (WGS) requires a considerable turnaround time and costs to provide results and may be insufficient for timely detection of variants for public health response, such as contact tracing and calculation of prevalence of VOCs in the community. Given the lack of possibility to implement WGS in all diagnostic laboratories in the era of the current pandemic, PCR-based diagnostic screening approaches that generate results in a few hours can be valuable [12].

The rapid emergence of novel variants clearly demonstrates the need for continued, robust and widespread SARS-CoV-2 surveillance and monitoring of the circulating SARS-CoV-2 variants. The aim of this study is to investigate the prevalence of SARS-CoV-2-positive samples bearing the N501Y mutation detected in a central COVID-19 PCR laboratory in Northern Cyprus.

## Materials & methods

### Sample collection & SARS-CoV-2 detection

Oro/nasopharyngeal swab samples were collected from individuals admitted to Near East University Hospital for various reasons, such as passengers entering Northern Cyprus from other countries and the individuals included in the population screening program according to the governmental SARS-CoV-2 management plan. Samples were stored in viral transport medium. A total of 658 samples collected between November 2020 and May 2021 were analyzed for the presence of SARS-CoV-2 using Diagnovital^®^ HS SARS-CoV-2 real time PCR kit (cat:09079100, RTA Laboratories Inc., Istanbul, Turkey) and Bio-Speedy^®^ Direct reverse transcription-quantitative PCR (RT-qPCR) SARS-CoV-2 kit (cat:BS-SY-SC2-1000, from Bioeksen R&D Technologies Inc., Istanbul, Turkey) following manufacturer’s protocols. RT-qPCR for the Diagnovital HS SARS-CoV-2 real time PCR kit was carried out at 45°C (20 min), 95°C (10 min) and 45 cycles of 95°C (15 s) and 58°C (45 s). For the Bio-Speedy Direct RT-qPCR SARS-CoV-2 kit, the program was 52°C (5 min), 95°C (10 s) and 40 cycles of 95°C (1 s) and 55°C (30 s). Insta Q96™ plus real-time PCR detection system (HiMedia Laboratories Pvt. Ltd, Mumbai, India) was used for RT-qPCR. Analysis was performed according to the manufacturer’s instructions and samples that have Ct (cycle threshold) values in the HEX channel were reported as positive for the SARS-CoV-2 presence.

The patient samples that were positive for SARS-CoV-2 were further investigated for the presence of N501Y mutation with reverse transcription quantitative PCR.

### RT-qPCR for detection of N501Y mutation

Hibrigen^®^ SARS-CoV-2 and N501Y mutation detection kit (Hibrigen Biotechnology R&D San Tic Ltd Sti., Gebze, Turkey) was used for the determining the presence of N501Y mutation in patient samples that tested positive for SARS-CoV-2. The kit contains primer and probe sets specifically designed for SARS-CoV-2 RdRp gene (FAM), nucleotide sequence harboring the N501Y mutation (HEX) and human RPII (Cy5) gene as an internal control. The reaction mixture was prepared following the manufacturer’s protocol. Briefly, 20 μl reaction mix was prepared with 10 μl of 2X One Step RT-PCR Mix, 4 μl of primer probe mix and 6 μl sample. A positive and negative control, which were provided by the kit, were included for each run. RT-qPCR was carried out at 55°C (15 min), 95°C (2 min) and 40 cycles of 94°C (10 s) and 63°C (20 s) using Insta Q96™ plus real-time PCR Detection System (HiMedia Laboratories Pvt. Ltd). Analysis was performed according to the manufacturer’s instructions and samples that have Ct values in the HEX channel were reported as positive for the N501Y mutation. Samples that were used in the study were from SARS-CoV-2-positive confirmed patients. As variant RT-qPCR analysis is a qualitative analysis method that aims to detect the presence or absence of the N501Y mutation, samples that had Ct values lower than 35 and had amplification curves in a sigmodial shape were accepted as ‘variant bearing’ according to the kit manufacturer’s instructions regardless of their Ct values

### Vaccination status of the population

The vaccination program has been implemented in Northern Cyprus since 15 January 2020, starting for the healthcare workers exclusively. Vaccination then began for the elderly over 65 years and were vaccinated starting from late February. It was not until February 2021 that vaccination was made available for the whole population. At this time period the only available vaccine was Sinovac CoronaVac. By the end of November 2021, 275,988 people were fully vaccinated with any of the available vaccines (two doses with Sinovac, Pfizer-Biontech and Astra-Zeneca, or single dose with Johnson & Johnson). 80,243 people are vaccinated with a third dose.

## Results

**Figure 1.**
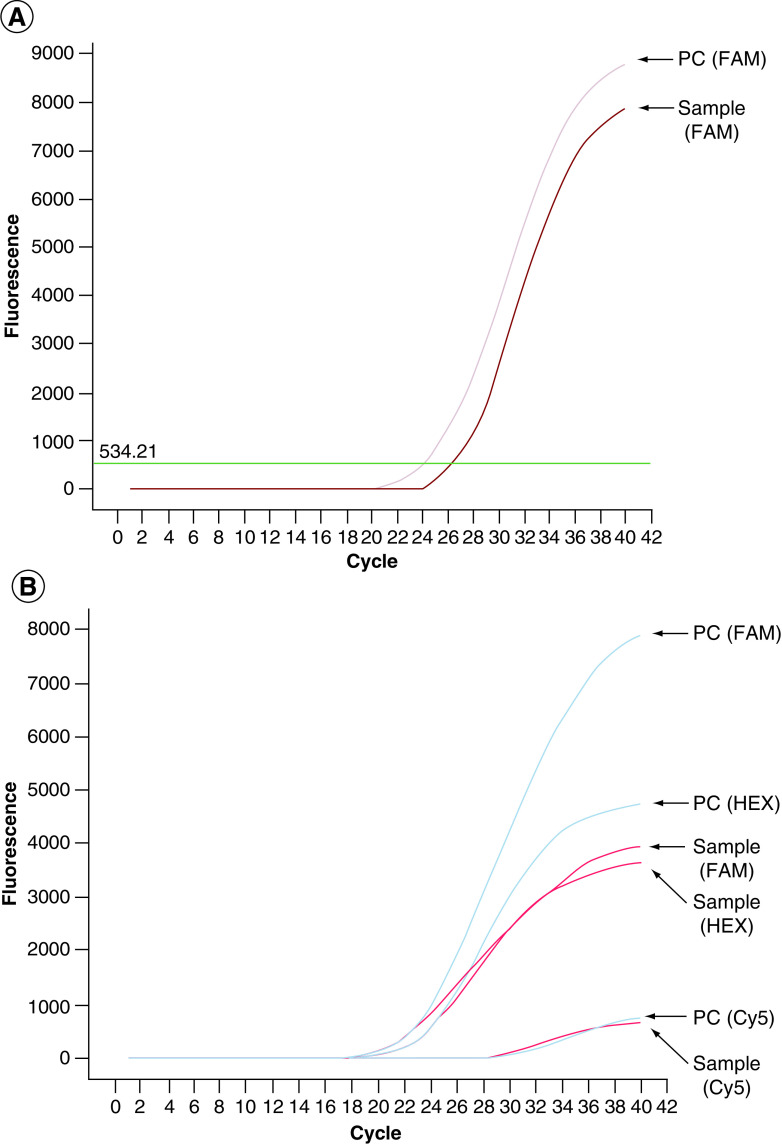
Amplification curve of a SARS-CoV-2-positive sample and positive control with the Diagnovital^®^ HS SARS-CoV-2 real time PCR kit is shown. **(A)** SARS-CoV-2-specific gene targets are detected in FAM channel. **(B)** Amplification curve of a SARS-CoV-2-positive sample with N501Y mutation and positive control with the Hibrigen^®^ SARS-CoV-2 and N501Y mutation detection kit is shown. SARS-CoV-2-specific gene targets are detected in FAM channel, N501Y mutation is detected in HEX channel and human internal control is detected in Cy5 channel.

A total of 658 SARS-CoV-2-positive samples were analyzed for the presence of N501Y mutation. Among these, the mutation was detected in 433 samples. A representative figure showing qPCR amplification curves of a SARS-CoV-2-positive sample and a sample with N501Y mutation is shown in Figure 1. In November 2020, N501Y mutation was not detected in any of the samples that were identified as SARS-CoV-2 positive (n = 4). Similarly, none of the SARS-CoV-2-positive samples (n = 13) were positive for the N501Y mutation in December 2020. The first SARS-CoV-2 sample carrying the mutation was detected in January 2021 in Northern Cyprus. Out of 31 SARS-CoV-2-positive samples, 14 (45.2%) were identified to have the N501Y mutation in January 2021. The presence of N501Y mutation was detected in 55 out of 90 (61.1%) samples in February 2021 and the frequency of the mutation continued to increase in March, April and May (69.2, 68.4 and 75.5% respectively).

It was observed that the variants with SARS-CoV-2 N501Y mutation increased significantly, while the variants without N501Y mutation decreased according to the months (p = 0.000; Table 1).

**Table 1.** SARS-CoV-2-positive samples not-bearing and bearing the N501Y mutation detected in our laboratories between November 2020 and May 2021 are shown in the table (n).

Month	SARS-CoV-2 N501Y negative, n (%)	SARS-CoV-2 N501Y positive, n (%)	Total (n)
November	4 (100%)	0 (0%)	4
December	13 (100%)	0 (0%)	13
January	17 (54.8%)	14 (45.2%)	31
February	35 (38.9%)	55 (61.1%)	90
March	41 (30.8%)	92 (69.2%)	133
April	90 (31.6%)	195 (68.4%)	285
May	25 (24.5%)	77 (75.5%)	102
Total	225 (34.2%)	433 (65.8%)	658

## Discussion

As the battle with the SARS-CoV-2 continues at different frontlines in healthcare systems and the scientific world, the pathogen acquires new mutations to adapt and survive, leading to the emergence of novel VOCs. Currently, several variants of the SARS-CoV-2 is in circulation around the world. Genomic analysis of the emerging variants is of high importance for the monitoring of the alterations in the viral genome which might have structural and functional effects that may potentially affect the severity of the disease, the diagnostic approaches and the preventive strategies. One of the persisting mutations detected in three important SARS-CoV-2 variants (B.1.1.7, B.1.351 and P.1) is the N501Y substitution in the spike protein amino acid sequence. Studies indicated that this modification in the receptor-binding domain of the virus enhance the affinity of SARS-CoV-2 spike protein to the host receptor, ACE2, increasing the transmissibility of the virus [13].

In this study, we aimed to investigate the frequency of SARS-CoV-2-positive samples harboring the N501Y mutation in Northern Cyprus. Considering the fact that the mutation can increase the rate of transmission, it is imperative to monitor the prevalence within the population. The data obtained in this study suggests that the variant with the N501Y mutation was initially observed in Northern Cyprus at the beginning of January 2021, as all of the samples dated November and December 2020 were lacking the mutation. The earliest samples that harbor the mutation were dated 2 January 2021. The frequency of SARS-CoV-2-positive samples with N501Y mutation increased rapidly from 45% in January to 61.1% in February and up to 75.5% in May 2021. Despite strict travel restrictions and compulsory PCR tests applied, a second wave of COVID-19 was observed in Northern Cyprus by February 2021. Daily cases increased from an average of 16.75–65.5 during this second wave. Total number of affected people, recorded since the first case was reported on 10 March 2020, had jumped from 1881 (15 January 2021) to 7229 (31 May 2021) according to the data shared at that time by the Turkish Republic of Northern Cyprus Ministry of Health. Increased transmissibility of the mutant virus was likely to contribute to the surge of COVID-19 cases in the country.

Genomic epidemiology studies performed in other countries including the USA, the UK and many European countries such as Portugal and Ireland similarly indicate that variants carrying N501Y mutation, in particular the B.1.1.7. variant of SARS-CoV-2, has rapidly became the dominant lineage which requires immediate and decisive action to minimize COVID-19 morbidity and mortality [14–16].

After the time of this study was completed, a new variant originated in India, later named as the Delta (B1.617.2) variant, and became the dominant variant. The Delta variant did not harbor the N501Y mutation, however Omicron (BA.1), which was defined as an emerging VOC and became the dominant variant worldwide in a very short time did harbor the N501Y mutation. This further suggest N501Y as an important mutation for increased transmission and survival of SARS-CoV-2.

The samples used in this study were obtained from a single laboratory, which can be a limitation. Further experiments including WGS of the SARS-CoV-2 samples isolated from patient oro/nasopharyngeal samples will enable us to have a better understanding about the variants in circulation.

## Conclusion

Monitoring the prevalence of important SARS-CoV-2 variations in circulation has been of great importance for surveillance studies. This study indicates that N501Y-bearing SARS-CoV-2 variants entered in circulation in Northern Cyprus at the beginning of January 2021 and rapidly spread to become the dominant variant in the population within 4 months. This increase in the prevalence of N501Y-bearing SARS-CoV-2 variants was in concordance with the increase in the number of positive cases per day, supporting previous studies suggesting enhanced transmissibility of these variants due to the N501Y substitution.

**Summary points**

- Since the start of the pandemic, several SARS-CoV-2 variants are being reported that could possibly lead to changes in transmissibility, clinical presentation and severity of the cases worldwide.
- UK (B.1.1.7, alpha), South Africa (B.1.351, beta) and Brazil (P.1, gamma) variants that were classified as variants of concern by the WHO contain the N501Y substitution mutation in the spike (S) gene.
- N501Y was previously shown to enhance viral binding to ACE2 host-receptor protein and increase transmissibility of variants.
- Aim of this study was to investigate the frequency of SARS-CoV-2-positive samples harboring the N501Y mutation in Northern Cyprus.
- Our study revealed that the variant with the N501Y mutation was initially observed in Northern Cyprus on 2 January 2021.
- The frequency of SARS-CoV-2-positive samples with N501Y mutation increased rapidly from 45% in January to 61.1% in February and up to 75.5% in May 2021. This was in correlation with a rapid increase in daily SARS-CoV-2-positive cases in the country.
- Our data support previous studies suggesting enhanced transmissibility of N501Y containing SARS-CoV-2 variants.
